# Rare and Imported Infections: Are We Prepared?

**DOI:** 10.3390/pharmacy7010009

**Published:** 2019-01-13

**Authors:** Godwin Oligbu

**Affiliations:** Paediatric Infectious Diseases Research Group, Institute for Infection and Immunity, St. George’s, University of London, London SW17 0RE, UK; godwin.oligbu@nhs.net

The world’s population is rapidly expanding. The current population of 7.7 billion is projected to increase by 1 billion over the next 12 years and reach 9.6 billion by 2050 according to the United Nations report. However, the global population is shrinking into a global village due to increasing intercontinental travel. International tourism has become one of the fastest growing sectors and most profitable sectors in the global economy through continuous expansion, diversification, and travellers increasingly pursuing new destinations, which often include remote areas of the world. In 2016, it was estimated that the number of trips constituting global international travel has risen to 1.2 trillion from 903 million in 2007, and compared to 25 million trips in 1950. A large proportion (i.e., 46%) of destinations include subtropical and tropical regions and an increase of 5% per year is predicted in travel to the Middle East, East Asia, and Africa [1]. For example, from 1987–2007, travel to the UK from all over the world was estimated to have been doubled from 16 million to 32 million visits. Among these, 4.5 million visits were from regions outside North America or Europe, and by 2016, over 19 million visitors stayed overnight in London [2]. Young children constitute 4% of overseas travellers, but make up nearly a quarter of all travel-associated admissions [3]. Considering the number of visits from UK residents, there was 65.7 million visits abroad in 2015 alone [1]. The number of visits to tropical countries has been increasing since 1996 at an average rate of 8% per annum [1], resulting in a global economic contribution of 7.6 trillion U.S dollars in 2016 [3]. With the current pattern of international travel, it is therefore estimated that over 280 million households globally will make at least one international trip per year by 2025 [4].

Apart from intercontinental travel, with recent natural disasters and displacement of people, including children, there is a global resurgence of hitherto controlled infections and the emergence of new ones with shift of existing diseases. Many of the most common infectious diseases, and particularly those transmitted by vectors, are prone to unstable environments and poor control of human environments. New and renaissance vector-borne communicable diseases, including arbo-viruses, such as dengue, Zika and Ebola, and malaria are widely evident [5]. Other infectious diseases, such cholera, have shown increased outbreaks in famine and highly deprived areas of the world, especially in camps.

Therefore, the risk of infection may be increased from one place to another. Notably, an infected returning traveller may not seek immediate healthcare support after returning to the UK or may delay this for days or weeks simply because symptoms and clinical signs may not develop for days or even weeks after his or her return, thus making it difficult to establish relationships between past travel and imported infections [6].

An imported infection would be defined as an infection of a returning traveller in which the incubation period is compatible with acquiring the infection abroad. A child in this context is 0 to 16 years of age. According to the European Union (EU), a rare disease is defined as one that affects less than 10 in 20,000 of the general population or disorder that affects less than 1 in 2000. Similarly, in the USA, a disease or disorder is said to be rare if it affects fewer than 200,000 Americans at any given time. In other words, it is therefore possible that a disease may be common in one part of the world but considered rare in another due to low prevalence of the disease in that region. Currently, there are between 6000 and 8000 known rare diseases and around five new rare diseases are described in medical literature each week. In the EU, as many as 30 million people may be affected by one of over 6000 existing rare diseases [7,8]. HIV/AIDS and Influenza A (H1N1) are examples of the most recent destructive global pandemics in history.

Recently, Public Health England (PHE) reported investigating an increase in cases of rare disease, particularly acute flaccid paralysis. So far in 2018, 28 cases have been reported to PHE. A rise has also been reported in the US. This disease has since been eradicated in Britain, the US, Australia and most part of Europe since 1988, with the advent of Sabin’s OPV vaccine in 1962 [9]. In addition, there has been an increase in zoonotic diseases, with the re-emergence of Lassa fever, first being reported in Nigeria and subsequently spreading across most parts of West Africa. Cases have been reported in some European countries such as Germany and Sweden. The risk of disease spreading to non-endemic countries by travellers from Lassa fever endemic countries in West Africa is very low. Widespread trade in animal husbandry among countries has also seen the rise in imported infections [10].

However, only a few studies have emphasised increasing frequency and changes in patterns of imported infections carried by travellers and related implications for the region, particularly in children [11,12,13]. A prospective study done by Andrew et al. with 58 children showed that the more common infections were malaria, traveller’s diarrhoea and hepatitis [13]. The travel history of patients with focus on the geographical area during the clinical history taking procedure is very significant in patient management and has wider implications for public health. The importance of travel history is reflected by British guidelines on the management and control of viral haemorrhagic fevers [14], which heavily rely on epidemiological evidence. Similar risk assessment algorithms should be employed regarding emerging infections in this current climate of changing disease transmission patterns, in order to understand the cause and predict the future impacts of these changes on the health of children.

Finally, healthcare workers are more likely to see an increasingly number of cases of rare and imported infections due to intercontinental travel, as well as human and environmental displacement. The principle aim of this special issue is to enhance a collaborative effort to share good practices amongst health care providers, especially in relation to uncommon and rare infections in children, as well as to provide evidence-based practice for learning and educational development, particularly of frontline health care professionals. This will undoubtedly include increased active surveillance activities, studies to elucidate the modes of indigenous transmission, and preventive measures.
